# Newspaper coverage of food insecurity in UK, 2016–2019: a multi-method analysis

**DOI:** 10.1186/s12889-021-11214-9

**Published:** 2021-07-09

**Authors:** Amy Yau, Hardeep Singh-Lalli, Hannah Forde, Matthew Keeble, Martin White, Jean Adams

**Affiliations:** 1 Centre for Diet & Activity Research, MRC Epidemiology Unit, University of Cambridge, Cambridge, UK; 2 Present address: Population Health Innovation Lab, Department of Public Health, Environments & Society, Faculty of Public Health & Policy, London School of Hygiene & Tropical Medicine, London, UK

**Keywords:** Food insecurity, Food poverty, Newspaper, Frequency analysis, Thematic analysis, Media analysis

## Abstract

**Background:**

Food insecurity is a growing concern in the UK. Newspaper coverage can reflect and shape public and political views. We examined how frequently food insecurity was reported on in UK newspapers, how the problem and its drivers were described, and which solutions were proposed.

**Methods:**

Using Factiva, we searched for news articles that were substantively about food insecurity and published in national UK newspapers between 01 January 2016 and 11 June 2019. We examined whether the number of articles differed over the study period, and conducted a thematic analysis to theoretical saturation using a random sample of articles.

**Results:**

Overall, 436 articles met our inclusion criteria and 132 (30%) were analysed thematically. Reporting was more prevalent in the summer, with mentions of ‘holiday hunger’ among children, and leading up to Christmas, when charity was encouraged. Articles often contained views from advocacy groups and charities, who appeared to play an important role in maintaining news interest in food insecurity. From the thematic analysis, we developed themes related to the problems (‘definitions of food insecurity’ and ‘consequences of food insecurity for individuals’), drivers (‘insufficient income as an immediate driver’ and ‘government versus individual responsibility’), and solutions (‘charitable food aid’ and ‘calls for government action’). The problem of food insecurity was often defined by food bank use or hunger, but other definitions and a range of consequences for individuals were acknowledged. Articles identified government as a driver of food insecurity, especially in relation to the roll-out of Universal Credit. Few articles proposed individual failings as a driver of food insecurity. The reported existing solutions predominantly focused on food banking and redistributing ‘food waste’. The public, charities, and individuals experiencing food insecurity were generally portrayed as supportive of government action to tackle food insecurity. However, contention within government regarding the extent of food insecurity, governmental responsibility and potential solutions was reported.

**Conclusions:**

Food insecurity was a topic of significant interest within UK newspapers. Newspapers were used to call for government action and advocate for structural, income-based solutions.

**Supplementary Information:**

The online version contains supplementary material available at 10.1186/s12889-021-11214-9.

## Introduction

Food insecurity can be defined as “the inability to consume an adequate quality or sufficient quantity of food in socially acceptable ways, or the uncertainty that one will be able to do so” [1]. The Food and Agriculture Organization of the United Nations highlights six dimensions of food security within their definition: food availability, food access, utilisation, stability, agency and sustainability [2]. Food insecurity has been identified as a growing problem in the United Kingdom (UK) [3]. We previously estimated that 24% of UK adults aged 18–64 years were living in food insecurity in 2017 [4]. Further, ‘holiday hunger’ (hunger among children during the school holidays, often due to the lack of free school meals) has been identified as a problem for children from low-income families [5]. Food insecurity is associated with poor diet quality and poor health, and likely contributes to health inequalities [4]. Whilst food insecurity can be a part of income poverty (often defined as households having less than 60% of the median income) and destitution (defined as lacking two or more bare essentials such as food, housing, heating, lighting, clothing, shoes and basic toiletries or extremely low income), the physical, psychological and social consequences of food insecurity warrant specific attention [6, 7].

Since 2010, a number of fiscal policies have been introduced in the UK as part of an austerity programme, resulting in welfare reform and funding cuts to social security and public services [8]. These measures were introduced by a centre-right Conservative (known colloquially as Tory) government. The Conservative government has been in power since 2010, first in coalition with the Liberal Democrats (politically central) and then as a single-party government from 2015 [9]. It has been proposed that these austerity measures have led to an increase in income poverty and income inequality in the UK [8]. Whether and how these political changes have led to an increase in food insecurity has been disputed [10]. Reports from The Trussell Trust (a charity that supports the UK’s largest network of food banks and has links to the church) and House of Lords Select Committee on Food, Poverty, Health and the Environment both suggested that the introduction of the Universal Credit welfare system was associated with a rise in food bank users, [11, 12] which is commonly used as a marker of food insecurity. These reports particularly pointed to the delay in receiving payment (of at least 5 weeks) when switching to the Universal Credit system. Universal Credit was introduced by the UK government to replace and unify six means-tested benefits [11]. The system is being rolled out gradually and is set to be in effect across the whole country by 2024 [13].

Public discourse in newspapers shapes and reflects public opinion [14]. News articles have previously been analysed to explore the framing of public health problems [15, 16]. Individuals experiencing food insecurity have reported feelings of stigma and shame [17]. This may be perpetuated by negative social attitudes and blaming of individuals within the public discourse [18]. Newspapers are also an important tool for public health advocacy, and can have substantial impact on the public and political agenda [14]. Different newspapers have, and are perceived as having, different political stances [19, 20]. Understanding attitudes towards food insecurity and the possible interventions that could be employed to reduce the prevalence, their feasibility and their potential acceptability across different actors (including governmental bodies, the general public, and various advocacy groups) may also help to inform how policies are implemented [21]. Therefore, analysing newspaper content on food insecurity could provide valuable insights on how to tackle the problem of food insecurity in the UK.

Two studies have previously investigated UK newspaper portrayal of food insecurity and food bank use. One study found no news articles reporting on food banks in 2007, and few before 2012 [22]. The number of articles increased dramatically between 2012 and 2014, corresponding with the increase in food bank usage [23]. Newspapers reported the emergence of tension between church leaders and The Trussell Trust, who cited changes to the welfare system as the reason for the increase in food bank usage, and the government, who attributed the rise in food bank use to the increased supply of food banks [10]. The absence of individuals directly experiencing food insecurity within newspaper discussions was also highlighted in this study. Another study that examined news articles on food insecurity in the UK published between 2006 and 2015 reported similar findings, adding that few articles specifically mentioned food insecurity among children and families [24]. This study also found that the majority of news articles were written in response to a specific event, report, or television programme (reactive reporting). Both studies noted that The Trussell Trust was a main actor cited in news articles on food insecurity. The studies also noted welfare reform as a driver mentioned within news articles from 2013, corresponding with the introduction of Universal Credit.

Whilst food insecurity remains prevalent, there are no studies on the newspaper coverage of food insecurity in the UK that have included articles published after 2015 [3, 4]. Furthermore, the ongoing roll-out of Universal Credit and austerity measures are likely to have exacerbated food insecurity, and more research into the drivers and consequences of food insecurity is warranted [11]. Thus, our study aimed to explore whether, and how, food insecurity was portrayed in UK national newspapers from 2016 onwards by examining the reporting frequency and the themes of published articles in terms of the proposed nature of the problem, its drivers, and solutions.

## Methods

We searched Factiva (https://global.factiva.com), an online database of media sources, for UK news articles related to food insecurity. We searched for uses of the words and phrases “foodbank”, “food bank”, “food insecur*”, “food poverty”, and “holiday hunger”, restricting articles to those published in the UK between 01 January 2016 and 11 June 2019 (the date when searches were conducted). We included all 12 national newspapers (*Independent, The Guardian, Daily Mirror, The Sun, The Times, Daily Express, Financial Times, Daily Star, Sunday People, Morning Star, Daily Mail* and *The Telegraph*) and retrieved articles from both daily and Sunday editions, and both those that were printed and published online.

The searches returned 2058 news articles. The Factiva database automatically removed some duplicates. AY removed remaining duplicates during the screening process, keeping the latest version of the article, or the longest version where multiple versions were published on the same day, based on the publication date and word count provided by Factiva. Articles with different headlines, but where over 80% of the content was the same, were treated as duplicates in the same way. News round-ups and summaries were excluded, assuming that more detailed articles would also be published alongside.

All remaining articles were assessed against inclusion and exclusion criteria (see Table 1) for eligibility by AY with duplicate screening by either HSL, HF, MK, or JA. All article types were eligible for inclusion, including letters from readers and editorials, but reader-generated online comments were excluded. Whether the topic of the article was ‘substantively’ about food insecurity was subjectively judged by the screeners based on the content of the article. Articles were classified as substantively about food insecurity if the screener judged food insecurity to be the main focus of the article. Discrepancies were resolved through discussion between the two original screeners and a third screener.

**Table 1:** Inclusion and exclusion criteria

	Include	Exclude
**Article type**	Articles that have had some editorial input (e.g. news articles, feature articles, letters from readers, opinions)	Reader-generated online comments
**Context**	Articles about real people and situations	TV listings or articles related to TV dramas, films, or fictional characters; articles about food insecurity in animals
**Topic**	Articles that are substantively about food insecurity – the problem, its drivers, or solutions	Articles that are not substantively about food insecurity i.e. mentioned in passing
**Country**	Articles about food insecurity in the UK	Articles about food insecurity in countries other than the UK
**Unit of measurement**	Articles about food insecurity at the household or individual level	Articles about food insecurity at the national or global level e.g. main topic is UK’s self-sufficiency in food production

To investigate patterns in the frequency of publication, we counted the number of included articles by newspaper title, political stance (left, centre or right), [19, 20, 25] and newspaper type (popular, mid-market or quality) (see Table 2) [26]. Whilst there is no agreed definition of ‘left’ and ‘right’ in politics, leftist ideology is sometimes described as progressive and tends to favour social equality, whilst rightist ideology is sometimes described as conservative or traditional and tends to favour liberty [27]. In this study, political stance of the newspapers was categorised based on public opinion, [19, 20] except for the *Morning Star*, where this information was not available and classification was based on self-categorisation from their website [25]. Newspaper type was based on the Audit Bureau of Circulations (ABC) classification, [20, 28] and is indicative of readership demographics, with readers of ‘quality’ newspapers more likely to have higher occupational level compared to readers of ‘popular’ newspapers [29]. We also identified the 10 months of our study period with the highest number of included articles and analysed the content of the articles published in these 10 months (*n* = 227) to explore whether the articles predominantly reported on a specific event, report, or topic – this process was data-driven.

**Table 2:** Classification of newspapers

	Category	Description
**ABC classification**	Popular	Tabloid newspapers aimed at a wide circulation
	Mid-market	Newspapers that are perceived as less populist than popular newspapers but smaller in format than broadsheet newspapers
	Quality	Newspapers that were traditionally^a^ broadsheet (~ 24 × 30 in.) and are perceived as high quality
**Political stance**	Left	Political ideology that is described as progressive and tends to favour social equality
	Centre	Political ideology that lies near the centre of the political spectrum
	Right	Political ideology that is described as traditional and tends to favour liberty

*ABC* Audit Bureau of Circulations.

^a^many have switched to a smaller format

All articles were imported to NVivo 12 Pro for qualitative analysis. We conducted a thematic analysis to explore how the problem of food insecurity, and its drivers and solutions, were presented within the articles, [30] using a blend of inductive and deductive approaches. A theme was defined as “stories about particular patterns of shared meaning across the dataset” [31]. AY developed the preliminary coding framework based on previous literature and an initial reading of the data. This coding framework was applied to a random 10% sample of articles to develop it further. The coding framework was then discussed and agreed with JA and MW. The agreed coding framework was applied to a further randomly selected 10% of articles and subsequently discussed and agreed with HF and MK, without further amendments. AY used the final, agreed framework to code articles in randomly selected 10% samples until thematic saturation (or theoretical sufficiency) was reached [32].

## Results

### Included articles

Of the 2058 articles screened, 436 (21%) met our inclusion criteria and were included in the quantitative analysis. The content of articles published in the 10 months with the highest number of included articles were analysed (*n* = 227) and a random 30% sample of all included articles were coded for thematic analysis (*n* = 132). Article length varied from 58 to 4811 words, with a median length of 608 words. The number of news articles included varied by newspaper (see Table 3). The *Independent* and *The Guardian* together were responsible for over half (55%) of the included articles. Overall, politically left-leaning newspapers accounted for 44% of articles, centralist newspapers for 32%, and right-leaning for 22%. Quality newspapers accounted for 62% of articles, mid-market for 4%, and popular for 30% [19, 20, 25].

**Table 3:** Number of included articles by newspaper title, political stance, and newspaper type

Newspaper	Number of articles (%)	Political stance	ABC classification
*Independent*	131 (30.0)	Centre	Quality
*The Guardian/Observer*	110 (25.2)	Left	Quality
*Daily Mirror/Sunday Mirror*	75 (17.2)	Left	Popular
*The Sun/Sunday Sun*	40 (9.2)	Right	Popular
*The Times/Sunday Times*	24 (5.5)	Right	Quality
*Daily Express/Sunday Express*	11 (2.5)	Right	Mid-market
*Financial Times*	11 (2.5)	Right	ND
*Daily Star*	8 (1.8)	Centre	Popular
*Sunday People*	8 (1.8)	ND	Popular
*Morning Star*	7 (1.6)	Left	ND
*Daily Mail/Mail on Sunday*	7 (1.6)	Right	Mid-market
*The Telegraph*	4 (0.9)	Right	Quality
**Total**	**436 (100)**		

*ABC* Audit Bureau of Circulations. *ND* no data.

### Quantitative analysis

**Fig. 1:**
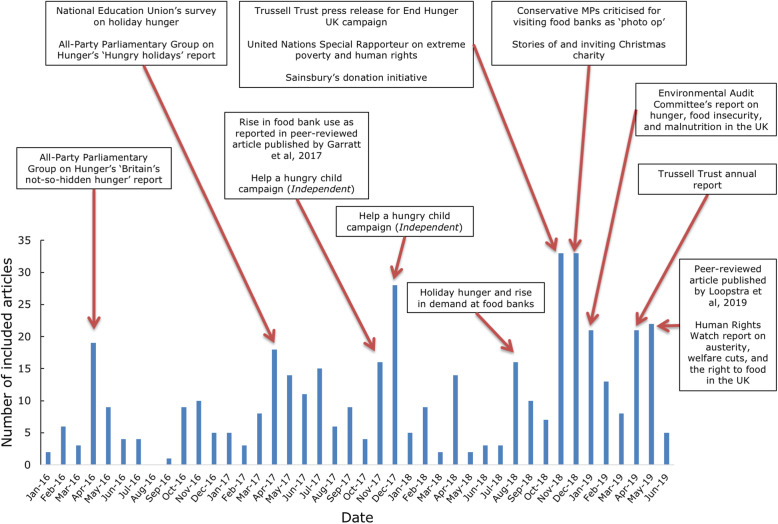
Number of included articles by month and the stories covered in the 10 months with the highest number of publications

The number of news articles substantively about food insecurity fluctuated by month (see Fig. 1). The 3 months with the highest number of included articles were in the run up to Christmas in December 2017 and November and December 2018. In 2017, the *Independent* ran the ‘Help a Hungry Child’ campaign in aid of the Felix Project, a charity that collects food that would otherwise be wasted and redistributes it to people experiencing food insecurity. In 2018, multiple groups encouraged Christmas charity to organisations supporting people experiencing food insecurity. These are examples of proactive reporting. Most other peaks resulted from reactive reporting and corresponded with the publication of reports, such as those published by The Trussell Trust (November 2018 and April 2019) and the All-Party Parliamentary Group on Hunger (April 2016 and April 2017). Both groups were previously identified as key actors in the newspaper discussion of food insecurity [22, 24].

### Thematic analysis

**Fig. 2:**
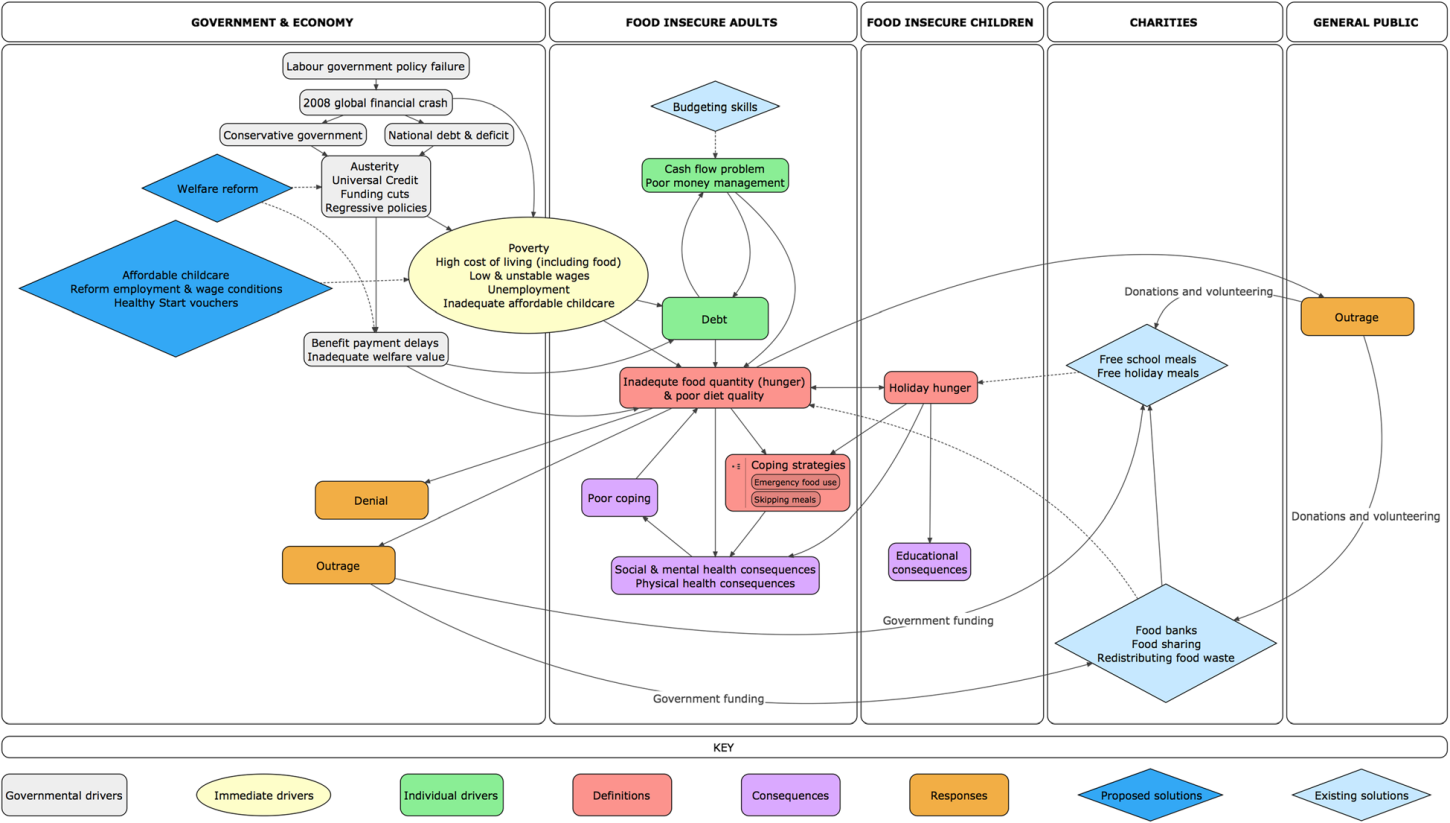
Conceptual map of the problem, drivers, and solutions of food insecurity as portrayed in UK newspapers, 2016–2019. Solid line = associations described in news articles. Dotted line = interventions that could provide solutions along the chain of events related to food insecurity

From the thematic analysis, we developed themes related to the problems (‘definitions of food insecurity’ and ‘consequences of food insecurity for individuals’), drivers (‘insufficient income as an immediate driver’ and ‘government versus individual responsibility’), and solutions (‘charitable food aid’ and ‘calls for government action’). Figure 2 is a conceptual map that represents how ideas within the identified themes were linked to each other as represented in the body of material analysed. Whilst some articles simply reported the latest statistics from recently published reports on markers of food insecurity, such as prevalence of food bank usage or the proportion of teachers reporting hunger among their students, some articles provide more in-depth reporting of the problem, its drivers, and solutions. This figure does not necessarily represent causal pathways, and some links are contested between different actors. Below we discuss each theme in turn, illustrating how our sample of news articles presented the relationships shown in Fig. 2. Where the quoted news article itself quotes an individual, we have identified the person by role or job title.

#### Problem of food insecurity

The themes related to the problem of food insecurity were ‘definitions of food insecurity’ and ‘consequences of food insecurity for individuals’.

##### Definitions of food insecurity

The problem of food insecurity was defined in several ways (illustrated in red in Fig. 2), with a focus on limited quantity of food available for children and adults. There was an emphasis on the timing of food insecurity for children, with reports of ‘holiday hunger’.

##### *Food insecurity as limited food quantity and quality*

The definitions of food insecurity we quoted in the introduction is more expansive than simply food insufficiency, citing six dimensions of food insecurity [1]. However, articles largely defined food insecurity as insufficient food quantity and by its symptoms, such as hunger and meal skipping:

*“Many have to skip meals and cut back on food to get by.”* (Deputy Director of Policy & Partnerships at Joseph Rowntree Foundation quoted in the *Independent*, 10 January 2019).

News articles also frequently presented food bank usage as synonymous with food insecurity and often used statistics on food bank usage to illustrate the severity of food insecurity in the UK:

*“Overall 1,109,309 emergency food packages were distributed by the Trussell Trust in 2015-16 – up slightly from last year. The charity, Britain’s leading food bank provider, said that the figure was ‘one million too many’ and urged the Government and the public not to accept the levels of food poverty in the UK as ‘the new normal’.”* (*Independent*, 15 April 2016)

Some articles made mention of diet quality, illustrated by consumption of typically ‘healthy’ foods such as fruit and vegetables:

*“The families of nearly 4 million children would struggle to afford enough fruit, vegetables and other healthy foods to meet the government’s nutritional guidelines”* (*Independent*, 05 September 2018)

##### *Food insecurity as holiday hunger in children*

The reporting of food insecurity among children was prominent in included articles. Holiday hunger was commonly used to define the extent of child food insecurity:

“*During the school term, almost all of these children are entitled to a free school meal. During the summer holidays, their families – already stretched to the limit – somehow have to find the money for those extra meals*.” (Convener of the Kirk’s Church & Society Council quoted in the *Sunday Express*, 09 July 2017)

The term ‘holiday hunger’ started to be used in news article headlines in April 2017, but mentions of hungry children and greater food insecurity in families with children during holidays were found in earlier articles in our sample.

##### *Food insecurity used to illustrate extreme poverty and destitution*

Despite excluding articles that mentioned food insecurity in passing (as a part of general poverty), poverty remained a prominent topic in included articles. Food insecurity and food bank usage were frequently used as illustrative of, and synonymous with, absolute poverty and destitution:

*“The Joseph Rowntree Foundation said it will urge Alston [UN rapporteur on extreme poverty and human rights] to examine how tougher benefit sanctions lead to greater destitution, which means people not being able to keep warm, fed, dry and clean. It found that last year 1.5 million people fell into destitution at some point – just over one in 50 people – with the highest levels in Manchester, Liverpool and Middlesbrough.”* (*The Guardian*, 22 August 2018)

##### Consequences of food insecurity for individuals

There was recognition within news articles that the consequences of food insecurity for individuals could be complex and varied (shown in purple in Fig. 2). Some articles mentioned mental health consequences:

*“Our research highlights that poor mental health is both a cause and a consequence of poverty. Of 20 food bank users we interviewed during one week, 18 said they had experienced poor mental health – stress, anxiety and depression – in the last 12 months. Six said they had considered or attempted suicide in the past year.”* (Trustee of Wandsworth Food Bank quoted in *The Guardian*, 11 May 2017)

Other consequences mentioned include the influence of food insecurity on social interactions and reluctance to seek help:

*“The food bank was a last resort for people who were mostly existing, not living, and this unsurprisingly led to stigma, shame, and embarrassment for many who were desperately trying to make ends meet.”* (Author of *Hunger Pains: Life Inside Foodbank Britain* quoted in *The Guardian*, 22 April 2016)

News articles on the impact of food insecurity on physical health often made reference to periods of British history renowned for poverty. Words such as “Victorian” or “Dickensian” were used, indicating an incompatibility with modern day living:

*“Health experts have warned of the return of Victorian scourges such as rickets and stunted growth due to child food poverty and malnutrition.”* (*Independent*, 19 December 2017)

For children, educational consequences were also cited frequently alongside a lack of future opportunities:

*“Scientific studies have found that [children experiencing food insecurity] lose one hour of learning time a day as a result of being distracted. This diminishes their school results, impacting on their chances for a good future.”* (*Independent*, 30 November 2017)

Articles also reported on how families distributed scarce food, with adults cutting back to ensure children did not have to, in order to cope with food insecurity:

*“46 per cent of mothers in the UK aged under 25 do not eat proper meals in order to ensure their children are fed, while more than a quarter have used food banks.”* (*Independent*, 28 March 2017)

#### Drivers

News articles reported on the immediate and upstream drivers of food insecurity. The immediate drivers centred on insufficient income (shown in yellow in Fig. 2), whilst debates over whether upstream drivers were governmental (shown in grey) or individual (shown in green) were reported.

##### Insufficient income as an immediate driver

Low wages and unstable incomes were frequently cited as reasons for food insecurity among the ‘working poor’:

*“We have 900,000 workers on zero-hours contracts not knowing when the next pay day will be*.” (Daily Mirror reader quoted in *The Daily Mirror*, 28 April 2017)

The high cost of living was also cited as a direct reason for food insecurity:

*“More than a quarter (28 per cent) of* [food bank users] *who had experienced rising expenses said this was due to housing costs, such as rent or energy, going up, with tenants in private housing were more likely to find it difficult to keep up with rents than socially rented properties.” (Independent,* 29 June 2017)

##### Government versus individual responsibility

Although there was apparent consensus in the data that the immediate drivers of food insecurity were low incomes and the high cost of living, thus resulting in insufficient income, there was disagreement as to whether these drivers were becoming more prevalent in the UK, and if so, why this was the case. The upstream drivers of food insecurity identified in the articles can be broadly categorised as governmental or individual.

##### *Governmental drivers: austerity and welfare reform*

Articles pointing to government policies as the drivers of food insecurity cited austerity policies following the global financial crash in 2008, which resulted in national debt and deficit: [33].

*“Wages stagnated and working conditions worsened under Tory [Conservative] austerity policies after the financial crash.”* (Guardian reader quoted in *The Guardian*, 19 November 2018)

Such government policies were portrayed as regressive (placing a greater burden on lower income rather than higher income groups) within articles, and therefore, exacerbating wealth inequality and driving food insecurity:

*“The root of the problem is our refusal to share the wealth we have more equitably. Instead of tackling inequality, we are pursuing policies that intensify it.”* (*Sunday Express,* 09 July 2017)

The design and implementation of the Universal Credit social security system, as part of the Conservative government’s welfare reforms, was particularly strongly linked to food insecurity in included articles:

*“Food banks handed out a record number of meals last year after the chaotic introduction of universal credit, the government’s flagship welfare overhaul, left claimants unable to afford meals when their benefits were delayed*.” (*The Guardian*, 25 April 2017)

The main problem with Universal Credit mentioned was the delay of initial payments – of at least 5 weeks – when switching from the old system. The “*harsh taper rate which punishes people for earning more*” (*The Sun,* 31 January 2019) was also considered problematic.

Politicians from the Labour party, the main opposition to the Conservative party, emphasised the government’s responsibility for tackling food insecurity and criticised the government’s actions:

*“‘No one should be cold or hungry at Christmas. It’s time this Government opened its eyes to the misery it is causing and immediately stop the roll out of Universal Credit.”* (Labour Leader (2015-2020) quoted in the *Daily Mirror*, 27 November 2018)

##### *Individual responsibility*

Although individual responsibility, such as money management, was mentioned in some articles, it was usually in a context where politicians were criticised for viewing food insecurity as individual failing:

*“The essence of poverty is not having enough cash or other resources to buy yourself out of these everyday problems of fluctuating incomes and needs which non-poor people can cope with. That is the fundamental difference between the Tory assertion that recourse to food banks is just a cash flow problem which anyone may have, and the reality of intensities of poverty which have not been seen in the UK since before the welfare state was introduced.”* (Co-founder of the Child Poverty Action Group quoted in *The Guardian*, 01 June 2017)

##### *Government response to food insecurity*

The Conservative government’s reported responses to food insecurity were varied. There was some denial – with arguments that food insecurity was not a problem in the UK, that the government had no role in causing food insecurity, and that it was not government’s responsibility to tackle food insecurity reported in included articles:

*“Household incomes have never been higher and the number of children living in workless households is at a record low, but we know there’s more to do ensure that every family has access to nutritious, healthy food. We already provide support through free school meals and our Healthy Start Vouchers, while we spend £90bn a year on working-age welfare and will be spending £28bn more by 2022 than we do now.”* (Government spokesperson quoted in the *Independent*, 10 January 2019)

When government representatives acknowledged the problem of food insecurity, they often supported charity as a solution whilst denying a link between government action and food bank usage:

*“Britain has a proud tradition of volunteering and of civil society and faith groups providing support to vulnerable people and this Government welcomes that. We know that the reasons for food-bank use are complex and often overlapping, so it is misleading to claim that it is driven by benefit delays. The vast majority of benefits are paid on time and improvements are being made year on year.”* (Government spokesperson quoted in the *Independent*, 03 January 2016)

Government officials attempted to demonstrate their support for charity by visiting food banks. This was met with cynicism and outrage within readers’ letters written into newspapers:

*“I feel absolutely incensed at the sight of Iain Duncan Smith and the rest of his smirking, smug, self-satisfied Tory cronies posing at foodbank collection points. He and the other Tory hypocrites are acting as if they’re Santa Claus or fairy godmothers rather than the Scrooges they really are.”* (Daily Mirror reader quote in *The Daily Mirror,* 18 December 2018)

Some Conservative politicians additionally pointed responsibility to their predecessors, the Labour party, who were in government from 1997 to 2010:

*“Inevitably, the state can’t do everything, so I think that there is good within food banks. The real reason for the rise in numbers is that people know that they are there and Labour deliberately didn’t tell them.”* (Conservative Member of Parliament quoted in *The Daily Express*, 15 September 2017)

Nonetheless, as scrutiny of the new welfare system increased, there was some government acknowledgement of problems with the Universal Credit system:

*“It is absolutely clear that there were challenges with the initial roll-out of Universal Credit. And the main issue that led to an increase in foodbank use could have been the fact that people had difficulty accessing their money early enough.”* (Welfare Secretary quoted in *The Daily Mirror*, 12 February 2019*)*

#### Solutions

Most of the solutions we identified were charity-based, but we also found some mention of solutions that were individual (skills-based) and structural. Solutions are illustrated within diamonds in Fig. 2. Solutions reported as currently existing are coloured light blue, and solutions that were proposed but are not enacted are coloured dark blue.

##### Charitable food aid

Existing solutions to food insecurity mentioned within articles were predominantly centred on charitable food aid. As found in previous studies, [22, 24] food bank usage dominated newspaper reporting of food insecurity. The redistribution of ‘food waste’ was also often mentioned as a solution within articles. Various initiatives were described where food that would otherwise end up in landfill was identified and used to feed people experiencing food insecurity. Given the environmental harms of food waste, these food redistribution initiatives were often described as a double win for both environment and food insecurity:

*“The charity [Felix Project] has been working since 2016 to fight hunger with surplus in-date produce, responding to the twin demons of food poverty and food waste. Now, it will be channelling all funds raised by The Independent’s [Help a Hungry Child] appeal to provide fresh and nutritious food for hungry children to access at market stalls in primary schools.”* (*Independent*, 07 December 2017)

Food waste redistribution was also described as providing an opportunity for the commercial sector to contribute to solving food waste and food insecurity:

*“Britain’s biggest supermarkets will commit to double the amount of surplus food they redistribute in a new drive to reduce waste.”* (*The Times*, 24 January 2017)

Some articles encouraged donations from the public and celebrated the success of charity projects, whilst others highlighted that charity initiatives were not sustainable and addressed the symptoms rather than the root causes of food insecurity. In some cases, the same organisation expressed both – superficially contradictory – views. The Trussell Trust was often quoted as advocating for structural understandings of food insecurity and government interventions:

*“The Trussell Trust’s chief executive, Emma Revie, said it was unacceptable that people had to use food banks in the first place, and the state should not rely on them to fix its shortcomings. ‘We do not want to be a part of the welfare state, we can’t be a part of the system.’”* (Chief Executive of The Trussell Trust (since 2018) quoted in *The Guardian*, 25 April 2019)

However, some articles reported that food banks were providing budgeting tips and cheap recipes alongside food aid to combat food insecurity. Their introduction of budgeting skills solutions suggests that they also see their role as providing interim support for individuals experiencing food insecurity:

*“As well as helping with problems relating to benefit payments and housing, the [food bank] advisers assisted people with managing their money and dealing with their debts. In the future a variety of types of assistance will be offered. McAuley says: ‘We’re calling it ‘money help’. In some places it won’t be financial advice – it will be budgeting skills.’”* (Chief Executive of The Trussell Trust (2014-2017) quoted in *The Guardian*, 30 January 2016)

##### Calls for government action

Within included articles, there were calls from charities, advocacy groups, and the general public for government action to address the perceived root causes of food insecurity – poverty and wealth inequality. In particular, there were calls for welfare reform to prevent delays to benefit payments, increase the value of benefits, and extend eligibility for welfare support:

*“Most of West Cheshire [food bank]‘s six recommendations on how to reduce the rising numbers of people dependent on its charity food handouts focus on welfare policy: more efficient jobcentre [UK government agency that helps people find employment] administration, a less punitive sanctions system, adequate levels of benefit payment, and a properly functioning local welfare safety net.” (The Guardian,* 22 July 2016)

Others called for improved workers’ rights, including ensuring that job contracts were secure and increasing the National Living (or Minimum) Wage [34]. Government policies that could ensure more flexible working options and affordable childcare for parents wishing to find employment were also mentioned:

*“Young mums have told us that they need better support from jobcentres, cheaper childcare, and flexible and part-time working opportunities to help them to find jobs and provide for their families. Now is not the time to be removing support for these young people, but to be helping them to build a fair financial future.”* (Chief Executive of the Young Women’s Trust quoted in *The Guardian,* 31 March 2017)

This would help food insecure families, especially young mothers (under 25 years) who are currently ineligible for some benefits and have lower wages [35].

The public response, found mostly in letters and opinion pieces, frequently captured outrage about both the problem and perceived drivers of food insecurity:

*“Families starving in our once-proud country is a disgrace. The Trussell Trust is doing an amazing job, as are other foodbanks around the country, but this is 2017 and as usual the rich get richer and tell those who struggle that they must just get on with it.”* (Daily Mirror reader quoted in *The Daily Mirror,* 28 April 2017)

Although the majority of actors (aside from government representations themselves) supported the notion of government-led action to address poverty, there was some support for the government’s current welfare system:

*“Universal credit is one of the most effective poverty-fighting tools in existence … when it is fully rolled out, hundreds of thousands more people will have a job as a result.”* (Head of Policy at the Centre for Social Justice quoted in *The Guardian*, 22 August 2018*)*

As illustrated in Fig. 2, governmental solutions are upstream of charitable solutions, and have the potential to address the perceived root causes of food insecurity and provide more sustainable solutions. However, reluctance from the government to take action to tackle food insecurity was portrayed throughout our study period, as demonstrated by the reported denial and lack of action, although some shifts in government response were noted.

## Discussion

### Summary of key findings

This is the one of the first studies to explore newspaper portrayal of food insecurity in the UK. Food insecurity was a topic of significant interest within newspapers. A high number articles about food insecurity in a given month usually coincided with an event or report (reactive reporting), with one notable newspaper-led Christmas charity campaign run by the *Independent* in 2017 (proactive reporting). Articles often contained views from advocacy groups and charities, who appeared to play an important role in maintaining news interest in food insecurity. Although there was a heavy reliance on food bank usage as the definition of food insecurity, we also found recognition of holiday hunger, poor diet quality, and reduced social participation among individuals experiencing food insecurity within articles. The development of a more nuanced understanding of food insecurity compared to that found in previous studies, [22, 24] was not reflected in the reported solutions, which relied heavily on food banks. Redistribution of food waste was popular in news articles as another charity-based solution. The perceived immediate drivers of food insecurity reported were low income and high cost of living, resulting in insufficient income. Whether and why these drivers have increased in the UK were disputed. The government’s reported responses ranged from denial of the problem and denial of responsibility to acknowledgement of a link between welfare reform (including the roll-out of Universal Credit) and food insecurity. There were calls from the general public and advocacy groups for government to address the perceived upstream drivers of food insecurity, including poverty and wealth inequality, with structural solutions.

### Strengths and limitations

This study provided an updated view on how food insecurity was portrayed in all 12 UK national newspapers from January 2016 to June 2019, a time period not included in previous studies [22, 24]. Furthermore, unlike the previous studies, we included letters and opinion pieces to capture how newspapers were being used as a channel of communication by the general public and advocacy groups. We also used a wider concept of food insecurity compared to previous work, where the focus was exclusively on food banks or a specific population subgroup. However, some limitations of this study should be noted. We randomly sampled articles to code for our thematic analysis. Theoretical saturation was reached after analysing 132 articles (30% of articles that met the inclusion criteria). By taking a random sample for analysis, we efficiently captured a large proportion of unique stories, as many articles within our sample covered the same stories. However, this approach might have been less able to capture potential variation in coverage of the same story between newspapers. Our inclusion criteria focused on articles that were substantively about food insecurity. This meant exclusion of many articles that used food insecurity as an example of poverty or destitution. However, food insecurity is a result of and a component of poverty, and the wider drivers and solutions may not have been captured fully in our work. Nonetheless, poverty, welfare reform, and the competing costs of living prevailed as common topics in included articles even with these exclusions. By including searches for ‘holiday hunger’ and ‘food banks’ specifically, we may have been more likely to discover news articles on these topics. Our search terms were based on searches conducted in previous studies and our knowledge of the wider food insecurity literature. However, there may have been areas that were omitted, and thus drivers and solutions that did not appear as strongly in our article sample as a result. Moreover, we only examined newspaper articles and did not look at other news sources, such as social media, which may also influence public perceptions.

### Comparison to previous studies

Similar to previous studies, [22, 24] we found that reporting on the rise of food bank usage was very common and many articles relied on food bank usage data to introduce the topic of food insecurity. However, we observed some recognition that food bank usage was not the only definition of food insecurity. Articles within our study contained some acknowledgement of the different dimensions of food insecurity [2]. Mentions of holiday hunger (stability), poor diet quality (utilisation), and reduced social participation among individuals experiencing food insecurity (agency) show a recognition of food insecurity as more than a food availability issue. Although reported solutions to food insecurity have also diversified beyond food banks, they still relied heavily on charity. Food waste redistribution has become a frequently mentioned solution. Unlike in previous work, [24] accounts from individuals experiencing food insecurity were common. Nonetheless, news articles remained largely reactive and often reported the latest statistics from reports by organisations such as The Trussell Trust and the All-Party Parliamentary Group on Hunger, which were found to be key organisations represented in newspapers previously [22, 24]. These organisations continued to have a strong advocacy presence within identified articles, and appeared to help maintain newspaper interest on food insecurity. Reports from other organisations, such as the United Nations human rights reports and the report by the Environmental Audit Committee, were also cited within articles in this study [36, 37]. In contrast to previous work, we found reporting on children and families experiencing food insecurity to be common [24]. This change could be due to some prominent reports and campaigns in 2017, including those by the All-Parliamentary Group on Hunger [38]. As in previous research, [24] we found few articles supporting the idea that food insecurity was a matter of failed individual responsibility. Articles mentioning individual responsibility in relation to food insecurity generally did so in a context of critiquing politicians for blaming individuals, suggesting that this was not in line with the views of the newspaper and its audience. Some articles reported that the government recognised food insecurity as a problem and one associated with the Universal Credit welfare system, which they previously denied [22]. Therefore, the drivers of food insecurity seem less contested than in previous studies.

### Interpretation and implications

The number of included articles fluctuated seasonally. More articles were published in the lead up to Christmas (observed in 2017 and 2018 in our sample). Christmas is a time of year that is traditionally associated with togetherness and giving [14]. The timing may be strategic to elicit emotion and encourage public support for charitable solutions. Although the British public seem to expect inequalities in health, wealth, and political power, [39] we found a strong sense of outrage associated with the existence of hunger in the UK. This could be important in a context where most solutions rely on volunteers and donations. The success of these interim solutions is necessary to provide temporary help for people experiencing food insecurity [40]. However, charitable food aid is not a long-term solution to food insecurity, as was recognised by charities themselves. The Trussell Trust, and similar charitable organisations, play an important dual role in providing short-term support for individuals experiencing food insecurity and advocating for long-term, structural solutions. These groups also appeared to play a crucial role in maintaining media interest in food insecurity in the UK and their voices were prominent in our sample of news articles. The recent focus on hunger in children within newspapers may be a deliberate strategy amongst advocacy groups to prioritise the problem and generate public support for structural solutions, as there is often specific protection afforded to children due to their perceived vulnerability [5, 41]. The similar reframing of obesity as a problem of childhood appears to have been a successful strategy to drive structural solutions in the UK [42, 43].

The focus on government responsibility within the included articles could be due to the majority of articles being published in left-leaning and centralist newspapers, particularly *The Guardian* and the *Independent*. The political ideology of these publications may mean that social problems are more likely to be viewed as structural rather than individual [27]. However, some structural solutions to public health issues in the UK have been introduced by a centre-right Conservative government, such as the Soft Drinks Industry Levy as part of the strategy to tackle obesity [43]. Left-leaning publications may also be more willing to criticise a centre-right government. Nonetheless, *The Sun, a* right-leaning newspaper, was responsible for 40 (9%) of included articles. The higher number of articles on food insecurity published in *The Sun*, compared to other newspapers, could be due to the demographic of the readership, who are likely to be younger and have lower incomes [29]. At the end of some included articles in *The Sun*, there was advice on “what to do if you have problems claiming Universal Credit”, suggesting that the publication recognised that some of its readers might be likely to be experiencing financial difficulties, and thus food insecurity. Similar advice was not seen in other newspapers.

Although the first UK food waste redistribution organisation (Crisis FareShare) was established in 1994, food waste redistribution was only noted as a theme in newspaper reporting of food insecurity from 2014 [24]. The increase in newspaper coverage of this potential solution since 2014, and continued interest beyond 2016 identified in this study, might reflect increasing environmental awareness, making simultaneously reducing food insecurity and food waste an increasingly attractive solution. This solution may also be considered more systemic than straightforward charitable solutions, and a sustainable way of address food insecurity.

Some articles stressed that government interventions to improve welfare support and employment policies were needed in order to achieve sustainable solutions to food insecurity. According to the included news articles, the government has largely in principle, and sometimes financially, supported charity initiatives as the solution to food insecurity instead. However, there has been some recent political interest in tackling food insecurity, with the Children’s Future Food Inquiry and the House of Lord’s Select Committee Inquiry on Food, Poverty, Health, and Environment looking into food insecurity in the UK [5, 44]. The National Food Strategy is also under development, and mentions the need to deliver healthy and affordable food to people regardless of where they live and how much they earn [45]. Further, there has been government commitment to measuring food insecurity annually in the Family Resources Survey, using the United States Department of Agriculture’s Food Security Survey Module – the most commonly-used measure in high-income countries [46]. With robust monitoring of the prevalence of food insecurity, targets to reduce food insecurity can be set and progress can be monitored. However, given the likely relationship between diet quality and food insecurity, there have been recommendations to incorporate measurement of food insecurity into a survey that focuses on diet, such as the National Diet and Nutrition Survey, instead [12].

## Conclusions

Food insecurity was a topic of interest within UK newspapers, particularly during the summer holidays and Christmas period. Articles often contained views from advocacy groups and charities, who appeared to play an important role in maintaining news interest in food insecurity. Newspaper reporting of food insecurity was dominated by talks of food banks, as previously found. However, in contrast to previous work, children have become a main focus within news articles, which could increase political will for action. Reported existing solutions to food insecurity rely on charitable food aid. Reporting on redirecting food waste to reduce food insecurity has become more common in news articles, perhaps appealing to those seeking more systemic solutions and those who are environmentally conscious. The government’s role in contributing to, as well as resolving, food insecurity in the UK was recognised by charities and members of the general public. Articles called for welfare reform and improved employment policies. Despite previous governmental denial, there has been some recent government acceptance of responsibility for the problem of food insecurity. Articles indicated a widespread belief that more structural changes are required to address food insecurity in the UK.

## Supplementary Information

**Additional file 1.**

## Data Availability

Not applicable.

## References

[1] Dowler E, Turner S, Dobson B (2001). Poverty bites : food, health and poor families.

[2] HLPE. Food security and nutrition: building a global narrative towards 2030. A report by the High Level Panel of Experts on Food Security and Nutrition of the Committee on World Food Security. Rome: 2020. www.fao.org/cfs/cfs-hlpe. Accessed 21 Apr 2021.

[3] Loopstra R, Reeves A, Tarasuk V (2019). The rise of hunger among low-income households: an analysis of the risks of food insecurity between 2004 and 2016 in a population-based study of UK adults. J Epidemiol Community Health.

[4] Yau A, White M, Hammond D, White C, Adams J (2020). Sociodemographic characteristics, diet, and health among food insecure UK adults: cross-sectional analysis of the international food policy study. Public Health Nutr.

[5] The Food Foundation. Children’s Future Food inquiry. London: The Food Foundation; 2019. www.foodfoundation.org.uk/. Accessed 16 Aug 2019.

[6] Joseph Rowntree Foundation. What is destitution? 2018. https://www.jrf.org.uk/blog/what-destitution. Accessed 11 Oct 2019.

[7] Joseph Rowntree Foundation. What is poverty? 2020. https://www.jrf.org.uk/our-work/what-is-poverty. Accessed 12 Jul 2020.

[8] Oxfam (2013). The true cost of austerity and inequality: UK case study.

[9] News BBC (2018). United Kingdom profile - timeline.

[10] The Trussell Trust. About - The Trussell Trust. 2019. https://www.trusselltrust.org/about/. Accessed 27 Nov 2019.

[11] Thompson E, Jitendra A, Rabindrakumar S (2019). #5weekstoolong: why we need to end the wait for universal credit.

[12] Select Committee on Food Poverty Health and the Environment. Hungry for change: fixing the failures in food. London: Authority of the House of Lords; 2020. https://committees.parliament.uk/publications/1762/documents/17092/default/.

[13] House of Commons Library. Constituency data: Universal Credit rollout. 2020. https://commonslibrary.parliament.uk/social-policy/welfare-pensions/benefits/constituency-data-universal-credit-roll-out/. Accessed 5 Jul 2020.

[14] Chapman S, Lupton D (1994). The fight for public health: principles and practice of media advocacy. First edit.

[15] Menashe CL. The power of a frame: an analysis of newspaper coverage of tobacco issues-United States, 1985-1996. J Health Commun 1998;3:307–325. https://www.tandfonline.com/doi/pdf/10.1080/108107398127139?needAccess=true. Accessed 16 Oct 2019, 4. 10.1080/108107398127139 10977260

[16] Hilton S, Hunt K, Langan M, Bedford H, Petticrew M (2010). Newsprint media representations of the introduction of the HPV vaccination programme for cervical cancer prevention in the UK (2005–2008). Soc Sci Med.

[17] Purdam K, Garratt EA, Esmail A (2016). Hungry? Food insecurity, social stigma and embarrassment in the UK. Sociology..

[18] Garthwaite K (2016). Stigma, shame and “people like us”: an ethnographic study of foodbank use in the UK. J Poverty Soc Justice.

[19] Mayhew F. How daily newspaper readers voted by title in the 2017 general election. Press Gazette 2017. https://www.pressgazette.co.uk/how-daily-newspaper-readers-voted-by-title-in-the-2017-general-election/. Accessed 9 Dec 2019.

[20] Smith M. How left or right-wing are the UK’s newspapers? YouGov. 2018. https://yougov.co.uk/topics/politics/articles-reports/2017/03/07/how-left-or-right-wing-are-uks-newspapers. Accessed 9 Oct 2019.

[21] Pell D, Penney T, Hammond D, Vanderlee L, White M, Adams J (2019). Support for, and perceived effectiveness of, the UK soft drinks industry levy among UK adults: cross-sectional analysis of the international food policy study. BMJ Open.

[22] Wells R, Caraher M (2014). UK print media coverage of the food bank phenomenon: from food welfare to food charity?. Br Food J.

[23] The Trussell Trust. Biggest ever increase in UK foodbank use: 170% rise in numbers turning to foodbanks in last 12 months. Salisbury: The Trussell Trust; 2013. https://trusselltrust.org/wp-content/uploads/sites/2/2015/06/BIGGEST-EVER-INCREASE-IN-UK-FOODBANK-USE.pdf. Accessed 16 Oct 2019.

[24] Knight A, Brannen J, O’Connell R, Hamilton L (2018). How do children and their families experience food poverty according to UK newspaper media 2006-15?. J Poverty Soc Justice..

[25] Morning Star. Morning Star | the People’s daily. 2019. https://morningstaronline.co.uk/. Accessed 19 Dec 2019.

[26] PAMCo. Newbrands: Individual Brand Reach. 2019. https://pamco.co.uk/pamco-data/data-archive/. Accessed 14 Oct 2019.

[27] White J. Left, right and beyond: the pragmatics of political mapping. LEQS Paper No. 24. 2010. 10.2139/ssrn.1624805.

[28] Mediatique. Department for Digital, Culture, Media & Sport Overview of recent dynamics in the UK press market. London; 2018.

[29] Ofcom (2018). News Consumption in the UK: 2018.

[30] Braun V, Clarke V (2013). Successful qualitative research: a practical guide for beginners. First edit.

[31] Braun V, Clarke V (2019). Reflecting on reflexive thematic analysis. Qual Res Sport Exerc Heal..

[32] Braun V, Clarke V (2019). To saturate or not to saturate? Questioning data saturation as a useful concept for thematic analysis and sample-size rationales. Qual Res Sport Exerc Heal.

[33] BBC News. Reality check: how big is the UK’s deficit and debt? 2017. https://www.bbc.co.uk/news/business-39897498. Accessed 16 Jan 2020.

[34] Davis A, Hirsch D, Padley M, Shepherd C, Davis A, Hirsch D (2018). A minimum income standard for the UK continuity and change and change.

[35] Young Women’s Trust (2017). What matters to young mums?.

[36] Alston P (2018). Statement on visit to the United Kingdom, by Professor Philip Alston, United Nations special rapporteur on extreme poverty and human rights.

[37] Environmental Audit Committee (2019). Sustainable Development Goals in the UK follow up: Hunger, malnutrition and food insecurity in the UK.

[38] Forsey A (2017). Hungry holidays: a report on hunger amongst children during school holidays.

[39] Howarth D, Marteau TM, Coutts AP, Huppert JL, Pinto PR (2019). What do the British public think of inequality in health, wealth, and power?. Soc Sci Med.

[40] The Trussell Trust. End of Year Stats - The Trussell Trust. 2019. https://www.trusselltrust.org/news-and-blog/latest-stats/end-year-stats/. Accessed 16 Aug 2019.

[41] UNICEF. The United Nations convention on the rights of the child. London; 2019.

[42] HM Government (2016). Childhood obesity a plan for action.

[43] Department of Health and Social Care (2018). Global Public Health Directorate: Obesity Food and Nutrition / 10800. Childhood obesity: a plan for action Chapter 2.

[44] House of Lords Select Committee. Food, Poverty, Health and the Environment Committee - UK Parliament. 2019. https://www.parliament.uk/food-pov-health-enviro-comm/. Accessed 6 Jan 2020.

[45] Department for Environment Food and Rural Affairs. Developing a national food strategy: independent review 2019 – terms of reference - GOV.UK. 2019. https://www.gov.uk/government/publications/developing-a-national-food-strategy-independent-review-2019/developing-a-national-food-strategy-independent-review-2019-terms-of-reference. Accessed 6 Jan 2020.

[46] Taylor A. Anna Taylor: why officially measuring the scale of food insecurity is crucial. 2019. https://blogs.bmj.com/bmj/2019/03/15/anna-taylor-why-officially-measuring-scale-food-insecurity-crucial/.

