# Supplementary material for: Newspaper coverage of food insecurity in UK, 2016–2019: a multi-method analysis
Source: BMC Public Health. 2021 Jul 9;21:1201. doi: 10.1186/s12889-021-11214-9 (PMC8268386; doi:10.1186/s12889-021-11214-9)
Supplement: Supplementary file 1 — Additional file 1. [file 12889_2021_11214_MOESM1_ESM.docx]

**Newspaper coverage of food insecurity in UK, 2016-2019: a multi-method analysis**

Amy Yau^1^, Hardeep Singh-Lalli, Hannah Forde*, Matthew Keeble*, Martin White, Jean Adams

Centre for Diet & Activity Research, MRC Epidemiology Unit, University of Cambridge, Cambridge, UK

*equal contribution

^1^Present address: Population Health Innovation Lab, Department of Public Health, Environments & Society, Faculty of Public Health & Policy, London School of Hygiene & Tropical Medicine, London, UK

Corresponding author

Amy Yau

Department of Public Health, Environments & Society

London School of Hygiene & Tropical Medicine

15-17 Tavistock Place

London WC1H 9SH

[amy.yau@lshtm.ac.uk](mailto:amy.yau@lshtm.ac.uk)

**Supplementary Table S1.** Number of included articles per year by newspaper

| Newspaper | 2016 | 2017 | 2018 | 2019 | Total |
| --- | --- | --- | --- | --- | --- |
| *The Independent* | 20 (27.8) | 58 (42.3) | 33 (24.1) | 20 (22.2) | 131 |
| *The Guardian* | 24 (33.3) | 26 (20.0) | 38 (27.7) | 22 (24.4) | 110 |
| *The Daily Mirror* | 11 (15.2) | 20 (14.6) | 31 (22.6) | 13 (14.4) | 75 |
| *The Sun* | 4 (5.6) | 11 (8.0) | 13 (9.5) | 12 (13.3) | 40 |
| *The Times* | 1 (1.4) | 6 (4.4) | 9 (6.6) | 8 (8.9) | 24 |
| *The Daily Express* | 3 (4.2) | 5 (3.6) | 2 (1.5) | 1 (1.1) | 11 |
| *Financial Times* | 5 (6.9) | 2 (1.5) | 4 (2.9) | 0 (0.0) | 11 |
| *Daily Star* | 2 (2.8) | 3 (2.2) | 2 (1.5) | 1 (1.1) | 8 |
| *Sunday People* | 1 (1.4) | 1 (0.8) | 4 (2.9) | 2 (2.2) | 8 |
| *Morning Star* | 0 (0.0) | 0 (0.0) | 0 (0.0) | 7 (7.8) | 7 |
| *Daily Mail* | 0 (0.0) | 4 (2.9) | 1 (0.8) | 2 (2.2) | 7 |
| *The Daily Telegraph* | 1 (1.4) | 1 (0.8) | 0 (0.0) | 2 (2.2) | 4 |
| Total | 72 (100.0) | 137 (100.0) | 137 (100.0) | 90 (100.0) | 436 (100.0) |

| **Key (by year)** |
| --- |
| Newspaper with the highest number of articles |
| Newspaper with ≥10 articles |
| Newspaper with ≥5 articles |
| Newspaper with ≥3 articles |
| Newspaper with ≤2 articles |
